# Diagnosis and treatment of Chiari malformation type 1 in children: the International Consensus Document

**DOI:** 10.1007/s10072-021-05317-9

**Published:** 2021-06-07

**Authors:** Luca Massimi, Paola Peretta, Alessandra Erbetta, Alessandra Solari, Mariangela Farinotti, Palma Ciaramitaro, Veronica Saletti, Massimo Caldarelli, Alexandre Casagrande Canheu, Carlo Celada, Luisa Chiapparini, Daniela Chieffo, Giuseppe Cinalli, Federico Di Rocco, Marika Furlanetto, Flavio Giordano, George Jallo, Syril James, Paola Lanteri, Christian Lemarchand, Martina Messing-Jünger, Cecilia Parazzini, Giovanna Paternoster, Gianluca Piatelli, Maria. A. Poca, Prab Prabahkar, Federica Ricci, Andrea Righini, Francesco Sala, Juan Sahuquillo, Marcus Stoodley, Giuseppe Talamonti, Dominic Thompson, Fabio Triulzi, Mino Zucchelli, Laura Valentini, International Experts - Jury of the Chiari & Syringomyelia Consensus Conference, “Milan, November 11–13, 2019”

**Affiliations:** 1 Pediatric Neurosurgery, Fondazione Policlinico Universitario A. Gemelli IRCCS, Largo A. Gemelli, 8, 00168 Rome, Italy; 2 Pediatric Neurosurgery, AOU Citta’ della Salute e della Scienza di Torino, Torino, Italy; 3 Department of Neuroradiology, Fondazione IRCCS Istituto Neurologico Carlo Besta, Milan, Italy; 4 Neuroepidemiology Unit – Scientific Directorate, Fondazione IRCCS Istituto Neurologico Carlo Besta, Milan, Italy; 5 Department of Neuroscience, AOU Citta’ della Salute e della Scienza di Torino, Torino, Italy; 6 Department of Pediatric Neurology, Fondazione IRCCS Istituto Neurologico Carlo Besta, Milan, Italy; 7 Pediatric Neurosurgery Division, Universidade Estadual de Londrina, Londrina, Brazil; 8 “Associazione Italiana Siringomielia e Arnold Chiari”, Garino, Italy; 9 Clinical Psychology Unit, Fondazione Policlinico Universitario A Gemelli IRCCS and UCSC, Rome, Italy; 10 Pediatric Neurosurgery, Santobono-Pausilipon Children’s Hospital, Naples, Italy; 11 Pediatric Neurosurgery Department, Université de Lyon, INSERM U1033, Hopital Femme Mère Enfant, Lyon, France; 12 Department of Neurosurgery, Fondazione IRCCS Istituto Neurologico Carlo Besta, Milan, Italy; 13 Department of Neurosurgery, Meyer Pediatric Hospital, Florence, Italy; 14 Institute for Brain Protection Sciences, Johns Hopkins All Children’s Hospital, St. Petersburg, FL USA; 15 Department of Pediatric Neurosurgery, Necker Enfants Malades Hospital, Paris, France; 16 Department of Diagnostic and Technology, Neurophysiopathology Unit, Fondazione IRCCS Istituto Neurologico Carlo Besta, Milan, Italy; 17 Conseil Scientifique APAISER S&C, Treillières, France; 18 Pädiatrische Neurochirurgie, Asklepios Kinderklinik, Sankt Augustin, Germany; 19 Department of Pediatric Radiology and Neuroradiology, Children Hospital V. Buzzi, Milan, Italy; 20 Department of Neurosurgery, Gaslini Children’s Hospital, Genoa, Italy; 21 Neurosurgery and Pediatric Neurosurgery, Vall d’Hebron Hospital Universitari, Neurotrauma and Neurosurgery Research Unit, and Universitat Autònoma de Barcelona, Barcelona, Spain; 22 Department of Neurology, Great Ormond Street Hospital for Children, London, UK; 23 Pediatric Neuropsychiatric Unit, AOU Citta’ della Salute e della Scienza di Torino, Torino, Italy; 24 Section of Neurosurgery, Department of Neurosciences, Biomedicine and Movement Sciences, University Hospital, Verona, Italy; 25 Department of Clinical Medicine, Macquarie University Clinical Associates, Sidney, Australia; 26 Department of Neurosurgery, ASST Niguarda, Milan, Italy; 27 Department of Neurosurgery, Great Ormond Street Hospital for Children, London, UK; 28 Department of Pathophysiology and Transplantation, Neuroradiology Unit, University of Milan, Fondazione IRCCS Cà Granda, Ospedale Maggiore Policlinico, Milan, Italy; 29 Neurochirurgia Pediatrica, IRCCS Istituto delle Scienze Neurologiche di Bologna, Bologna, Italy

**Keywords:** Chiari 1 malformation, Syringomyelia, Consensus, Children, Craniovertebral decompression, Management

## Abstract

**Background:**

Chiari malformation type 1 (CM1) is a rare condition where agreed classification and treatment are still missing. The goal of this study is to achieve a consensus on the diagnosis and treatment of CM1 in children.

**Methods:**

A multidisciplinary panel formulated 57 provisional statements based on a review of the literature. Thirty-four international experts (IE) participated in a Delphi study by independently rating each statement on a 4-point Likert scale (“strongly disagree,” “disagree,” “agree,” “strongly agree”). Statements that were endorsed (“agree” or “strongly agree”) by < 75% of raters were re-formulated, or new statements were added, and another Delphi round followed (up to a maximum of three).

**Results:**

Thirty-five IE were contacted and 34 agreed to participate. A consensus was reached on 30/57 statements (52.6%) after round 1. Three statements were added, and one removed. After round 2, agreement was reached on 56/59 statements (94.9%). Finally, after round 3, which took place during the 2019 Chiari Consensus Conference (Milan, Italy), agreement was reached on 58/59 statements (98.3%) about four main sections (Definition and Classification, Planning, Surgery, Isolated Syringomyelia). Only one statement did not gain a consensus, which is the “definition of radiological failure 24 month post-surgery.”

**Conclusions:**

The consensus document consists of 58 statements (24 on diagnosis, 34 on treatment), serving clinicians and researchers following children with CM1. There is a clear need for establishing an international network and registry and to promote collaborative studies to increase the evidence base and optimize the long-term care of this patient population.

## Introduction

Chiari malformation type 1 (CM1) has gained a great interest among the scientific community because of the continuously increasing number of diagnoses and several controversial issues, especially about definition, management, and outcome assessment. The literature is richer and richer of papers, but in spite of some meta-analyses [1–6] and some international surveys [7–10], it still fails to achieve consensus as far as both methods and results are concerned. Moreover, no guidelines have been provided so far, although they are claimed by patients, Patients Associations, and physicians. These problems have to be faced both in affected children and adults.

This paper reports and comments on the results of the International Consensus Conference held in Milan in November 2019 with regard to the pediatric population. The goal of this meeting was to find a consensus among international experts on the most controversial issues to clear the way for creating guidelines.

## Methods

The results of the literature review (published in a separate article of the same issue) emphasized the necessity of a consensus strategy among the CM1 community. The parents of CM1 children, the Parental Associations, and the clinicians taking part in the diagnostic and treatment process further confirmed this need. Therefore, a panel of experts of the Chiari and Syringomyelia Consortium [11] formulated 57 draft statements on the main and controversial topics concerning CM1 and syringomyelia. A group of international experts (IE), from within and without Europe, designed by scientific production and by the Patients Associations, and with a cumulative experience of more about 8800 children (more than 3500 out of them were operated on), were asked to take part in the study as jury panelists.

The statements were distributed in a general addendum and in two separate questionnaires, one concerning pediatric patients and the other one adult patients. A Delphi process [12] was designed to reach a consensus of at least 75% of agreement. Three Delphi rounds were considered adequate as a reasonable effort to reach consensus among the experts. IE received a structured document to be voted on a 4-point Likert-type scale (“strongly disagree,” “disagree,” “agree,” “strongly agree”). In each round, IE indicated their level of agreement on the proposed statements. Statements that did not gain consensus (<75% of IE voting “agree” or “strongly agree”) were redistributed to participants in rounds 2 and 3.

## Results and discussion

This paper is focused on the results of the pediatric questionnaire and on the part of the general addendum addressing pediatric issues.

**Fig. 1:**
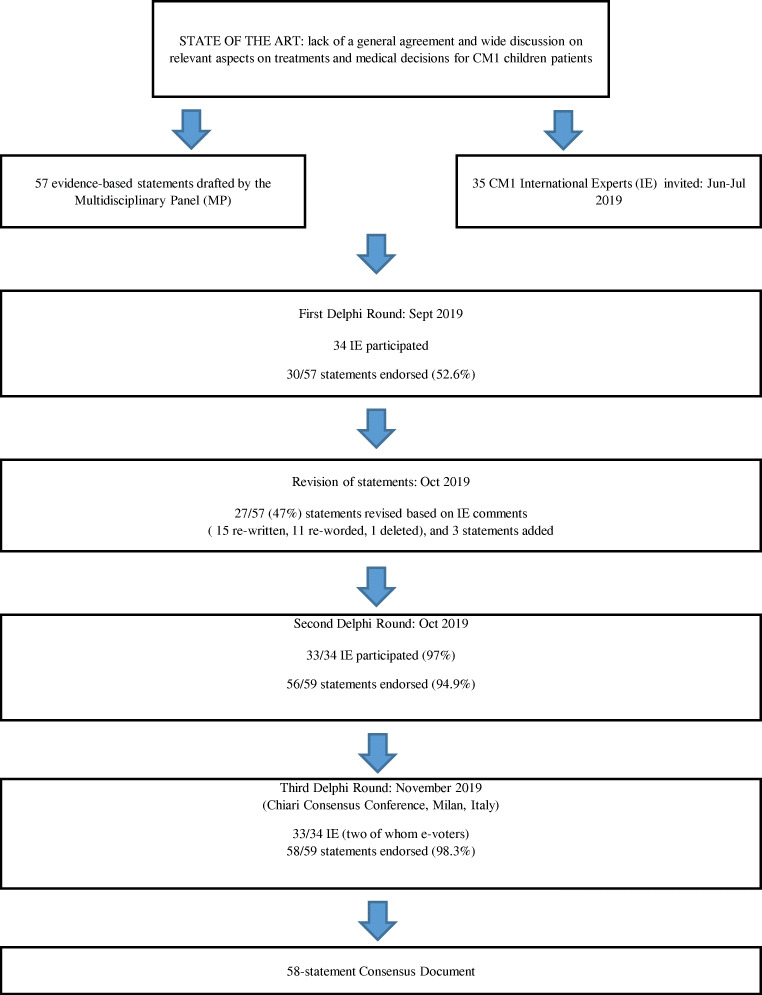
Flowchart of the Delphi study

**Fig. 2:**
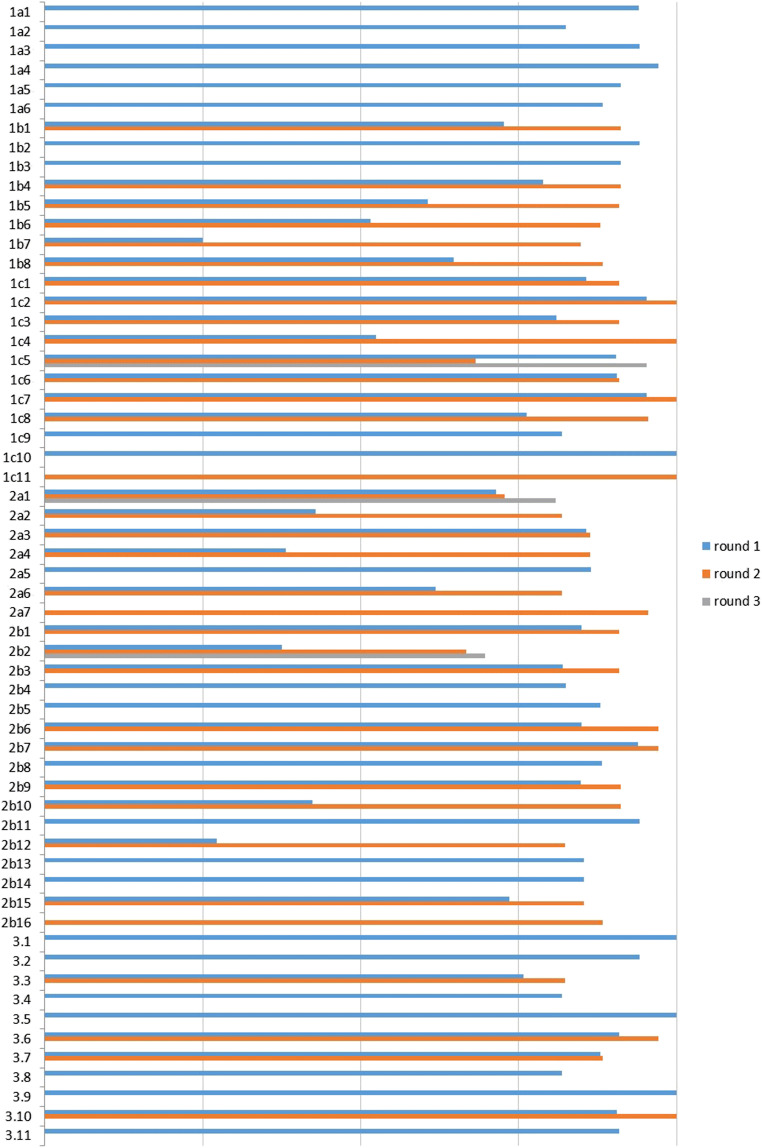
Agreement (%) on each statement of the Children Consensus Document in the three Delphi rounds (35 experts)

Thirty-five international experts (IE) were contacted and 34 agreed to participate in the Delphi process. The IE jury comprised 21 neurosurgeons, five neuro-radiologists, four pediatric neurologists, one neurologist, one psychologist, and two members of Patients Associations. A consensus was reached on 30 statements (52.6%) after round 1. Subsequently, three statements were added and one removed. After round 2, agreement was reached on 56 statements (94.9%). The final discussion and voting took place during the Chiari Consensus Conference held in Milan, Italy, November 2019, with a 98.3% of final agreement (Fig. 1). Only one statement, regarding the definition of radiological failure after surgery at 24 months follow-up, remained under the requested agreement percentage (Fig. 2).

### Classification and definition

This consensus conference arose from the ongoing work of an international task dedicated to the radiological classification and definition of CM1 and syringomyelia. An initial priority was to ensure that the panelists shared a common language. The agreement on the classification was as high as 85%, which was considered adequate for the Consensus. The results are reported in detail in this issue in the article “Diagnosis and Treatment of Chiari Malformation and Syringomyelia in Adults: The International Consensus Document.”

In the absence of a better radiological definition, CM1 was considered herniation of one or both cerebellar tonsils ≥ 5 mm below McRae’s line or even 3 to 5 mm but with syringomyelia or peg-like appearance of the tonsils. The transient tonsillar herniation resulting from hydrocephalus or other sources of mass effect is not considered CM1 but acquired tonsillar ectopia. Therefore, both the acquired tonsillar ectopia resulting from intracranial hypotension and intracranial hypertension should be ruled out with appropriate examinations, which are clinical pattern and contrast-enhanced MRI for CSF hypotension and clinical pattern, fundoscopy, and venous angio-MRI (or even direct ICP measurement, if needed) for raised ICP. Cough headache, signs and symptoms of brainstem and/or cerebellum and/or spinal cord dysfunction, otoneurological symptoms, and scoliosis remain the main findings composing the Chiari syndrome [13].

Syringomyelia and syringobulbia refer to longitudinally oriented fluid-filled cavities of any size respectively in the spinal cord and brain stem, expanding from the region of its largest diameter in the upper and/or lower direction. However, the definition, diagnosis, and classification of syringomyelia/syringobulbia still raise many questions as not every fluid-filled cavitation of the spinal cord deserves the diagnosis of syringomyelia and the distinction between syringomyelia and central canal dilatation may be difficult [14–16]. Pending a better radiological definition and etiopathogenic classification, a classification distinguishing 4 types of syringomyelia is still used, type I-a being associated with CM1.

Patients with CM-I and syringobulbia/syringomyelia may have relevant symptoms because of the brainstem and spinal involvement. The most frequent syringobulbia-related symptoms, usually in a chronic pattern, are headache, gait and balance disorders, limb weakness or dysesthesia, IX and X cranial nerve dysfunction, persistent hiccup, oscillopsia, nystagmus, Horner syndrome, and central hypoventilation syndrome [17]. The typical clinical symptoms of syringomyelia consist of dysesthesia, sensory disturbances, or pain caused by maneuvers, such as Valsalva, cough, laughter, or sneezing in the corresponding dermatomes, while motor deficits tend to be a late symptom. A centrally positioned syrinx typically causes dissociated sensory loss, while a syrinx involving the posterior horns causes neuropathic pain, often described as a burning type of constant pain aggravated during resting periods [15, 18, 19]. Hydromyelia (thin, centrally located dilatation of the spinal cord) is usually found to be asymptomatic.

#### Section 1—Planning for CM1 in children

##### Table 1 summarizes the results of Part A: Indications to surgery

This section strongly confirms conservative management in asymptomatic children without syringomyelia, even in case of significant tonsillar descent on MRI. Such an attitude already emerged in previous surveys, provided by the Pediatric Section of the American Association of Neurological Surgeons (2000) [8], by the International Society for Pediatric neurosurgery (2004, 2018) [9, 10], and by the Brazilian Society for Pediatric Neurosurgery (2020) [7]. This policy is reinforced by the rarity of abrupt and severe (post-traumatic) onset in previously asymptomatic patients [20–22]. The presence of syringomyelia is frequently considered the most important criteria for surgery in an asymptomatic patient as long as the syrinx is thicker than 5–8 mm or progressively enlarging. The cut-off (> 5 mm or > 8 mm) is still debated, and there is little evidence to correlate syringomyelia size with the presence of symptoms. However, the progression of syringomyelia seems to be more straightforward. The appearance of symptoms remains the main indication of surgery regardless of the size of the syrinx.

**Table 1:** Planning for CM1 in children: indications to surgery

1	In *asymptomatic* children with incidentally discovered, isolated CM1 and *no syringomyelia***,** surgery is not indicated	Agreement: 94.1%
2	In asymptomatic children with incidentally discovered CM1 and syringomyelia, surgery is indicated in cases of *syrinx larger than 5–8 mm*, and smaller syrinx increasing in size	Agreement: 82.4%
3	In children with *epilepsy* and CM1, surgical treatment of CM1 does not improve the seizure disorder	Agreement: 94.1%
4	In children with CM1 and *cognitive* and/or *behavioral* disorders (such as autism) not able to correctly report their symptoms because of communication impairment, careful clinical and instrumental assessments are mandatory to detect symptoms and signs of cerebellar tonsils ectopia	Agreement: 97.1%
5	In children with CM1 and cognitive and/or behavioral disorders (such as autism) and no clear CM1 symptoms, surgery is not indicated to improve the clinical picture	Agreement: 91.2%
6	Surgical treatment is not decided on *MRI pattern* in asymptomatic children with tonsillar ectopia ≥ 20 mm without syringomyelia	Agreement: 88.2%

There is also a consensus that Chiari surgery should not be offered to children with CM1 and epilepsy as a means of improving seizure control. The high agreement on this topic results from both daily clinical practice and literature, based on the evidence that the association is random [23, 24]. Similarly, there is not an indication to operate on the CM1 in children with autism or other cognitive/behavioral disturbances since there is no evidence yet of correlations between the two conditions and, in particular, no evidence that the treatment of CM1 improves the behavior disorder [25–28]. However, in this subset of patients, a careful clinical and instrumental assessment is mandatory because the affected children may not be able to accurately report CM1 symptoms and clinical signs may be hard to detect. On these grounds, autistic children who are symptomatic for CM1 and who undergo successful surgery can show an improvement in their cognitive and behavioral performances, but this results from the reduction of pain or other misdiagnosed symptoms. Some reports investigating the role of genetic mutations in isolated cases showed that the association between CM1 and autism and/or epilepsy is occasional, except for very rare and specific syndromes [29–32]. According to the experience of some panelists, the ventral compression of the basilar artery (due to dens malposition) may overt seizures or cognitive deficits related to raised ICP.

##### A synopsis of the results of Part B: Symptoms and follow-up is reported in Table 2

This section addresses the problem of the follow-up in asymptomatic subjects and the management of symptoms in the symptomatic ones. As far as the first group is concerned, there is agreement on the follow-up children with an incidental diagnosis of CM-1 until the end of their growth. Actually, although CM1 and syringomyelia tend to remain silent in the majority of asymptomatic children, a clinical and/or radiological progression cannot be excluded in a minority of them [33–36]. Although some examples of schedule are quite widely accepted (e.g., controls at 1, 3, and 5 years), the clinical and radiological check-up should be tailored according to each patient. If MRI is planned, it should include both the brain and whole spinal cord.

**Table 2:** Planning for CM1 in children: symptoms and follow-up

1	In *asymptomatic* children with incidentally discovered CM1 and *no syringomyelia*, neurological follow-up and whole neuraxis MRI should be performed until the end of growth, with a schedule based on clinical picture and degree of tonsils herniation.	Agreement: 91.2%
2	*Neuro-pediatric evaluation* is mandatory in all children with CM1 and *headache*	Agreement: 94.1%
3	Neuro-pediatric evaluation is mandatory in all children with CM1 and *symptoms* and/or signs of the brainstem, cerebellar, and/or cervical cord dysfunction to identify co-pathologies	Agreement: 91.2%
4	Neuro-pediatric evaluation is mandatory in all children with CM1 and *cognitive* and/or *behavioral disorders*	Agreement: 91.2%
5	Somatosensory evoked potentials (*SEPs*) may be used in CM1 children only in association with other tools (both clinical and instrumental)	Agreement: 90.9%
6	Auditory evoked potentials (*BAEPs*) may be used in CM1 children only in association with other tools (both clinical and instrumental)	Agreement: 87.9%
7	Motor evoked potentials (*MEPs*) may be used in CM1 children only in association with other tools (both clinical and instrumental)	Agreement: 84.8%
8	Polysomnography is indicated in very young children (< 6 years), in case of suspected apneas, and in case of severe cerebellar tonsils descent	Agreement: 88.2%

In symptomatic children, the role of the pediatric neurologist seems to be crucial in achieving a correct indication to surgery. Firstly, it is important for the differential diagnosis between CM1 headache and migraine or headaches resulting from other conditions. Inadequate and inaccurate assessment of headache will result in a wrong indication for surgery [37, 38]. Several studies have attempted to address this topic and proposed criteria to correctly diagnose the Chiari headache (cough headache, Valsalva headache, occipital or suboccipital, lasting a few minutes) [39, 40]. Moreover, a careful neurological evaluation is mandatory to rule out comorbidities and to correctly understand complex children (such as those with behavior disorders).

The use of neurophysiological examinations (SSEPs, MEPs, AEPs) is not routine but should be driven by clinical and/or radiological criteria. These should not be considered firstline examinations and their role in the diagnosis and evaluation of CM1 is unproven. Additionally, in spite of their utility in case of brainstem or spinal cord involvement, their role in the intraoperative monitoring at the moment of positioning and during the posterior fossa decompression remains controversial [41, 42]. Polysomnography is indicated to confirm the diagnosis of sleep apneas at any age, in particular in infants and small children with significant overcrowding of the posterior fossa. The occurrence of sleep-disordered breathing in children with CM1 is well-documented [43–45], and even a certain correlation between radiological and polysomnographic picture has been found [46].

##### Part C: Associated malformations: the results are summarized in Table 3

There was general agreement that, when present, hydrocephalus should be treated in the first instance reserving foramen magnum decompression for cases where symptoms persist in spite of adequately controlled ICP. The treatment of hydrocephalus is addressed first to restore the CSF pathways and to relieve from raised ICP (which is often also the main source of symptoms), thus limiting the risks of complications (cerebellar ptosis, pseudomeningocele) in case of following posterior fossa decompression [47]. Moreover, it has been demonstrated that many children undergoing the treatment for hydrocephalus by endoscopic third ventriculostomy show a significant improvement of the CM1/syringomyelia and so do not require any specific therapy for CM1 and the associated syrinx [48, 49].

**Table 3:** Planning for CM1 in children: associated malformations

1	In symptomatic children with CM1 and hydrocephalus, it is recommended to treat hydrocephalus first to relieve raised intracranial pressure and to avoid post-operatory complications. CM1 can be treated afterwards if symptoms do not disappear	Agreement: 90.9%
2	In CM1 children with non-syndromic craniosynostosis (sagittal or lambdoid synostosis, oxycephaly), the craniosynostosis is better treated before the CM1	Agreement: 100%
3	In infants with syndromic craniosynostosis and CM1, the surgery should first increase the cranial volume, with the proper vault remodeling for each syndrome	Agreement: 90.9%
4	In case of persistent CM1, in children with syndromic craniosynostosis already submitted to cranioplasty, the surgical approach depends on the syndrome, cranial volume obtained by previous operations, posterior fossa volume, and CM1 symptoms/associated syringomyelia	Agreement: 100%
5	In CM1 toddlers with polisynostosis and prevalent brachycephaly (and in selected cases in the other age classes), a posterior vault osteogenic distraction could be considered before performing a posterior fossa decompression	Agreement: 95.2%
6	Angio-RM or angio-CT is mandatory to rule out anomalous subcutaneous drainage before craniovertebral decompression in CM1 children with syndromic craniosynostosis	Agreement: 90.9%
7	CM1 is rarely associated with dysraphisms*and tethered cord syndrome (*tethering of the medulla at any level due to split cord malformation, limited dorsal myeloschisis, retained medullary cord, terminal myelocystocele, conus lipomas, thickened, and fatty filum with a low-lying conus (below L3))	Agreement: 100%
8	In the rare occurrence of CM1 in children with tethered cord syndrome, the de-tethering procedure should be performed to prevent neuro-urological deterioration and has no influence on CM1.	Agreement: 95.2%
9	The association of CM1 with “occult tethered spinal cord” (a tethering syndrome with a specific urodynamic pattern with a conus normally positioned above L2) has never been demonstrated.	Agreement: 81.8%
10	The intradural section of the filum terminale in CM1 children is recommended just to treat tethered cord syndrome and it plays no role in the management of a possible CM1 syndrome.	Agreement: 100%
11	The extradural section of the filum terminale in CM1 children is not recommended either to treat tethered cord syndrome or for the management of a possible CM1 syndrome.	Agreement: 100%

Because of their etiopathogenetic role in acquired CM1, non-syndromic craniosynostoses (mainly oxycephaly but also posterior lambdoid and sagittal synostosis) have been universally assumed to be treated before than CM1, whose management should be considered only in case of persistent symptoms and clear resolution of the synostosis picture. It is worth reminding that the rate of symptomatic CM1 subjects with undiagnosed craniosynostosis (namely, scaphocephaly) can be surprisingly high (15.5%) [50].

The same considerations apply to syndromic craniosynostoses, where the restoration of a proper cranial volume plays an important role. The association between syndromic craniosynostosis and CMI represents a complex interaction between intracranial volume, venous hypertension, disordered CSF circulation, and skull base abnormality. The management of CM1 in this instance needs to be evaluated in the wider context of craniofacial management. This is best dealt with by specialist craniofacial teams. There is a high rate of clinical and radiological recurrence in this subset of patients, and in situations of clinical/radiological persistence of CM1, a new operation should be carefully weighted according to the syndrome and the results of the previous cranioplasty/craniofacial distraction. Should the CM1 picture be clearly predominant, it can be addressed directly if the intracranial hydrodynamics have been adequately optimized. The posterior osteogenic distraction, proposed by some authors in children with recurrent CM1 [51], should be limited to patients with synostotic brachycephaly. In these children, the posterior distraction is likely to cure both the craniosynostosis and the associated CM1. Should a posterior fossa decompression be needed in syndromic children, a careful preoperative workup based on angio-RM is mandatory to rule out anomalous venous drainages and for a correct surgical planning [52].

The association between CM1 and the tethered cord is frequently debated in clinical practice and in the literature mainly because of the postulated “occult tethered cord syndrome” [7]. We found strong consensus on this topic: (1) the association between CM1 and the “true” or “manifest” tethered cord is universally found to be very rare and occasional [53]; (2) in those rare instances where a tethered cord syndrome is present in CM1 subjects, a de-tethering procedure is successfully performed to cure the syndrome but does not produce effects on the CM1 [54]; (3) the occult tethered cord syndrome remains a controversial and questionable entity [55] and more than 80% of panelists questioned its existence; (4) in case of symptomatic tethered cord, an intradural section of the filum terminale is recommended. The patient and his/her family should be aware that this operation is not addressing the associated CM1 [56, 57]; (5) the international experts were not aware of any evidence to support the use of the extradural section of the filum for either tethered cord syndrome or CM1.

#### Section 2—Surgery for CM1 in children

##### The results of Part A: Surgical techniques are reported in Table 4

The most appropriate surgical paradigm to treat pediatric CM1 is one that reflects the etiological heterogeneity of this condition. Once predisposing factors such as hydrocephalus have been dealt with, foramen magnum decompression remains the mainstay of treatment. In the literature, there are a large number of meta-analyses addressing the relative merits of bone-only decompression of the posterior fossa and decompression with duraplasty [1–6]. Most of these articles recently appeared (last 5 years) and report on a large number of patients, sometimes composing purely pediatric series or, more often, mixed series. Almost invariably, the comparison between the two techniques shows that the bony decompression alone has a very low rate of complications but a lower success rate; conversely, the duraplasty ensures better results but with a higher rate of complications (namely, the CSF-leakage-related ones). Accordingly, bony decompression is suggested in children without syringomyelia (and without severe symptoms), taking into account a possible risk of symptom recurrence. On the other hand, duraplasty is preferable when syringomyelia (and marked symptoms) is present, taking into account a certain risk of complications. The agreement on this is high but not complete (80%), because some panelists prefer to perform the most radical treatment as the first step (duraplasty with or without tonsil coagulation) to avoid re-operations and to favor a quick resolution of the syringomyelia, while others do not accept the surgical risk of duraplasty and acknowledge the low improvement provided by the bony decompression alone. Several panelists suggested the use of intraoperative ultrasounds to determine the need for dural opening (duraplasty in case of suboptimal CSF flow and tonsils pulsations). The only prospective study available on this topic (82 adolescents, 40 treated by bony decompression and 42 by duraplasty) shows a similar clinical outcome and syrinx improvement between the two techniques, with longer operation times and hospital stay and higher risk of complication by duraplasty [58]. Subsequently, many panelists highlighted that the strategy “syringomyelia = duraplasty” should not be assumed as an absolute rule. Therefore, a small or an asymptomatic syrinx can be successfully managed by bony decompression alone, while a clearly symptomatic or a recurring one could require even a IV ventricle stent according to some authors.

**Table 4:** Surgery for CM1 in children: techniques

1	In *symptomatic CM1 children without syringomyelia*, the bony decompression of the posterior fossa alone could be performed for the low complication rate, if the family accepts the perspective of possible second surgery	Agreement: 80.9%
2	In CM1 children with syringomyelia, bony decompression + duraplasty is preferable	Agreement: 81.8%
3	The extent of the bony decompression of the posterior fossa should be wide on the foramen, always including C1 laminectomy, and never extended to C2 for the risk of CVJ instability.	Agreement: 86.4%
4	In CM1 without arachnoiditis, it is indicated to preserve the arachnoid membrane to avoid CSF leakage and delayed scarring	Agreement: 86.4%
5	Cerebellar tonsils coagulation/resection is indicated in cases of very low-lying tonsils and recurrent or residual syringomyelia	Agreement: 86.4%
6	Autologous and allograft dural patches are preferable to artificial graft; both are suitable, according to the surgeon’s preference, while there are experiences suggesting to avoid the artificial ones	Agreement: 81.8%
7	A watertight suture helps preventing CSF leakage, by non-resorbable stitches, together with a strict muscle and soft tissue closure.	Agreement: 95.5%

A higher agreement (86.4–95.5%) was reached in respect of the technical details. For bony decompression alone, the crucial region to be decompressed is that involving the bulbo-cevical region, which is represented by the foramen magnun and the C1 posterior arch that should be opened with resection of the transverse dural band at this level. The occipital squama should not be opened widely, to avoid the cerebellar ptosis; furthermore, extensive muscle strip and C2 laminectomy should be avoided where possible to reduce the risk of craniovertebral instability. Once again, the extent of the bone opening and its landmarks should be tailored to the characteristics of each patient. In the case of duraplasty, the subarachnoid space should not be violated (unless arachnoiditis is present) because this maneuver increases the risk of CSF leakage and arachnoid scarring. Similarly, the subpial coagulation of the tonsils should be limited to very low tonsillar ectopia (below C2) and/or recurrent syringomyelia and/or severe hindbrain dysfunction, since this maneuver adds some morbidity other than a risk of arachnoid scarring. A crucial point in the case of duraplasty is the dural closure, which should be capacious and watertight at the same time, possibly with non-resorbable stitches to ensure a longer endurance. In this context, both autologous grafts and allografts are suggested as a good option rather than artificial grafts. Although the latter are burdened by a higher risk of specific complications (foreign body reaction, systemic immune response, premature graft dissolution, high costs), their use is widespread [59–61]. The technique of the closure is more important than the materials used to do it.

##### Part B: Surgical outcomes (Table 5)

This section addresses one of the most heterogeneous aspects of the CM1 management, which is the definition and the assessment of the surgical outcome. A standardized method to evaluate the postoperative outcome for CM1 has not been widely embraced [62]. In clinical practice, the time to declare failure and the clinical and radiological criteria for failure may vary significantly in the different centers and, sometimes, among the members of the same center. Therefore, it is not surprising that this issue was the only point of “disagreement” (agreement < 75%) registered among the panelists during the whole vote process. In spite of this, the definition of clinical surgical failure was widely accepted (90.9%), being characterized by the persistence of symptoms 12 months after surgery. Such a period is usually sufficient to observe an improvement even after a bony decompression of the posterior fossa. The clinical evaluation is suggested also as a mandatory step for the early assessment of outcome (6 and 12 months from the operation) together with brain and spinal cord MRI. In this phase, cine-MRI is regarded as helpful but not necessary in all cases. Instead, as mentioned, a sufficient agreement was not reached about the radiological definition of surgical failure. Actually, although the persistence of CM1 and syringomyelia 24 months after surgery seems a reasonable criterion, several panelists refused to separate the radiological from the clinical assessment. Moreover, a certain disagreement arose in defining time to failure, with one-half of panelists suggesting to re-operate a child for persistence of unchanged syrinx after 12 months (the stability of the syrinx was judged as a good outcome by the remaining panelists). As a result, the consensus was reached only when the definition of failure needing re-operation included worsening/persistence of clinical signs and/or symptoms, judged as the best way to assess the surgical outcome. Such a conclusion seems to conflict with the indication for surgery in asymptomatic, large syringomyelia (see Section 2, part A, question 2) and encourages (1) to define MRI criteria for failure, inclusive of functional studies of CSF spaces, and (2) to collect large multicenter series evaluated by comparable outcome scales.

**Table 5:** Surgery for CM1 in children: outcomes, failure, re-intervention

**Surgical outcomes**		
1	The early surgical outcome is assessed at 6 and 12 months by clinical evaluation and whole neuraxis MRI; a cine-MRI may be helpful.	Agreement: 90.9%
2	Surgical failure is clinically defined by the persistence of Chiari symptoms 12 months post-surgery.	Agreement: 90.9%
3	Surgical failure is radiologically defined by persistence of low tonsils and unchanged syringomyelia at 24 months post-surgical MRI.	Agreement: 69.7%
**Diagnosis and treatment of the main causes of surgical failure**		
4	Insufficient bone decompression is one of the causes of failure, it is diagnosed by 3D CT scan and the treatment is widening its extension.	Agreement: 82.4%
5	Posterior fossa arachnoiditis is one of the causes of failure: its treatment is adhesiolysis and/or tonsil resection; possible adjunctive procedures may be stenting of the IV ventricle with perimedullary space.	Agreement: 87.9%
6	Postoperative CSF leakage is a predisposing factor for infections and surgical failure due to arachnoiditis.	Agreement: 97.1%
**7**	An intracranial hypertension (IIH) may be a cause of failure. When intracranial hypertension is present, angio-MRI for venous study and 3DCT scan should be performed to exclude other causes of raised ICP such as pseudotumor, hydrocephalus, or craniosynostosis.	Agreement: 97.1%
8	A CVJ instability is one of the causes of failure. To diagnose it, a dynamic CVJ study is indicated, especially in CM 1.5 patients.	Agreement: 88.2%
**Surgical re-intervention**		
9	In case of symptomatic CSF leakage, a new operation is necessary.	Agreement: 91.2%
10	In case of asymptomatic CSF collection, conservative management is indicated as the first option.	Agreement: 91.2%
11	In case of unresolved/increasing CSF collection despite conservative treatment, a diagnostic re-evaluation is necessary to decide on the surgical option.	Agreement: 94.1%
12	*Asymptomatic children* without syrinx with persistent tonsil descent should be followed up for at least 24 months to monitor the appearance of clinical symptoms.	Agreement: 82.4%
13	*Asymptomatic children with persistent syrinx* at MRI at 12 months follow-up should be strictly followed up, and *re-operated* if scoliosis and/or symptoms occur.	Agreement: 85.3%
14	Children with *persistent symptoms* and unchanged MRI (no tonsils ascent and absent flow) at 6 or 12 months follow-up should be re-operated on.	Agreement: 85.3%
15	Children with *persistent symptoms and syringomyelia* at MRI at 6 or 12 months follow-up should be re-operated on.	Agreement: 85.3%
16	In case of *success of surgery*, the long-term postoperative follow-up is performed by a clinical examination and whole neuraxis MRI for at least 10 years, or until the end of growth, with a timetable depending on clinical and MRI patterns.	Agreement: 88.2%

##### Part C: Diagnosis and treatment of the main causes of surgical failure (Table 5)

Apart from the wrong indication to surgery and the occurrence of complications (surgeon-related causes), some patient-related causes of failure have been reported in some series, such as comorbidities, unfavorable or complex anatomy, tonsils below C2, diameter of the spinal cord, and pediatric age (if compared with adulthood) [1, 63–65]. As far as the technique is concerned, the main cause of failure of the bony decompression alone is judged to be the too small bone opening/bone regrowth, especially at the level of the foramen magnum [66, 67]. In selected cases (where MRI is not clear enough), 3D CT scan may be helpful in estimating the extent of the decompression and in planning a new operation to enlarge the bone opening.

As far as duraplasty is concerned, incomplete expansion duraplasty, arachnoid scarring, and wound-related complications are the main factors negatively affecting the outcome [65, 67]. Postoperative arachnoiditis is regarded as the most important cause of clinical and/or radiological recurrence. It can be addressed by a revision surgical procedure to perform a lysis of the adherences and/or to coagulate the tonsils (to gain space) or, in case of multiple recurrences, to stent the IV ventricle to maintain the patency of the obex [68]. The role of the IV ventricle stenting is still under debate.

Majority agreement was achieved regarding comorbidities which increase the risk of failed surgery independently from the used technique. Failure to appreciate and address important causative factors for Chiari such as raised intracranial pressure and craniovertebral instability is a predominant cause of treatment failure. The most feared complication is CSF leakage (which, though very rarely, can occur also after bony decompression alone) that is considered a predisposing factor for arachnoiditis (especially because of the resulting infective or aseptic meningitis). Raised ICP, on the other hand, is a possibly misdiagnosed cause of failure. If suspected, it should be ruled out clinically (fundoscopy, clinical history) and radiologically (angio-MRI in case of presumed pseudotumor cerebri, CT scan in case of craniosynostosis) to avoid unnecessary re-operations on CM1/syringomyelia. Similarly, an underestimated CVJ instability is likely to cause persistence of symptoms after surgery. Its role in the clinical picture should be properly addressed by clinical assessment and by a dynamic radiological study.

##### Part D: Surgical re-intervention (Table 5)

This part was devoted to the management of the complications/comorbidities presented in part C and the failed surgery. The first 3 questions addressed the management of CSF leakage, which was differentiated between symptomatic (open CSF leak or painful, closed subcutaneous CSF collection) and asymptomatic (subcutaneous CSF collection that does not cause symptoms or esthetic impact). The true CSF leakages and the symptomatic CSF collections deserve an aggressive management, which is a surgical revision of the duraplasty and the surgical cavity (plus external lumbar drainage, if needed). On the other hand, a conservative management (diuretic therapy, steroids, bandage, wait and see) is advised in case of asymptomatic “closed” CSF collections. However, should the latter persist for a long time or increase, a new diagnostic work-up and, possibly, a re-operation have to be considered.

As mentioned, the radiologic persistence of tonsils ectopia is not felt to be a failure. Therefore, if asymptomatic and without syringomyelia, children with persistent radiological CM1 should only be followed up to rule out the appearance of symptoms. In the panelists’ opinion, the minimum follow-up for recurrence of symptoms is 2 years after surgery but, more properly, it should be protracted till the end of the patient development. The same policy is advised for asymptomatic children with persistent syringomyelia (which means persisting at least 1 year after surgery). A new surgical operation is carried out in case of occurrence of symptoms or scoliosis. Scoliosis is actually found to be an active sign of CM1 with/without syringomyelia in several cases [69, 70].

A significant agreement was reached also about children with persistent symptoms and missed radiological improvement (CM1 and/or syringomyelia persisting 6 or 12 months after surgery): they should be re-operated on. Six months can be proposed as the minimum time to wait before the declaration of failure (to be shortened if the patient is severely symptomatic), while 12 months is the maximum one.

Finally, children who underwent a successful surgery are advised to be followed up until the end of growth or at least for 10 years, clinically and radiologically (at least one brain and spinal cord MRI), to exclude recurrences. The frequency of the follow-up controls should be scheduled through a personalized plan.

##### Part E: Surgical options for specific conditions related to CVJ malformations (Table 6)

A separate section was dedicated to the CVJ instability because of its still unclear relationship with CM1 and the still missing general agreement on its management, which is often different and even independent from CM1 [71–73]. Particularly controversial is the use of fixation to manage CM1 patients without CVJ anomalies [74].

**Table 6:** Specific conditions related to CVJ malformations: surgical options

1	CM 1.5 may be associated with basilar invagination or impressio basilaris. Only cases with related symptoms need to be operated.	Agreement: 95.2%
2	The preferred, etiological, surgical option for symptomatic basilar invagination associated with CM1 without atlanto-axial instability, could be anterior decompression, when posterior reduction has already failed.	Agreement: 85.7%
3	The preferred surgical option for basilar invagination with atlanto-axial dislocation is posterior fixation.	Agreement: 100%
4	Craniovertebral junction (CVJ) instability is a mobile dislocation between C0, C1, and C2 (according to neuro-radiological exams) leading to neuro axial compression, neurological deficits, progressive deformity, or structural pain.	Agreement: 85.7%
5	The standard diagnostic work-up for CVJ instability in CM should include (other than MRI) dynamic X-rays + dynamic CT scan with 3D reconstructions.	Agreement: 81%
6	CVJ fixation, with or without posterior decompression, is not indicated in CM1 patients without a documented CVJ instability.	Agreement: 90.5%
7	Posterior decompression and CVJ fixation is the preferred surgical option for CM patient with CVJ instability and related symptoms.	Agreement: 90.5%
8	In order to identify the best surgical option for CVJ instrumentation in a CM patient it is mandatory to identify the following: (A) the vertebral artery course by preoperative neuro-radiological studies (Angio-MRI, Angio-CT) and (B) the bone thickness of the occipital crest, the C2 isthmus diameter, the volume of C3 lateral masses.	Agreement: 90.5%
9	To identify the best surgical option of C1–C2 instrumentation it is mandatory to define the following: (A) the vertebral artery course by preoperative imaging (angio-MRI, angio-CT) and (B) the C2 isthmus diameter	Agreement: 85.7%
10	Fixations by C0-C3 or C1-C2 in CM patients with CVJ instability should be decided on the basis of local anatomy.	Agreement: 95.2%

A first, large agreement was achieved about the need to operate on only symptomatic subjects with basilar invagination/impression basilaris-related CM 1.5. This result is apparently obvious, but in clinical practice, a certain trend to propose prophylactic surgery emerged (probably due to the more severe radiological picture). As far as symptomatic basilar invagination without atlanto-axial instability is concerned, the proposed option is to attempt a posterior reduction first and, in case of failure, to perform an etiologic treatment by anterior decompression (followed by posterior fusion). The option of the posterior reduction first, as observed by the panelists, allows also to rule out microinstability and to verify if the odontoid is reducible, thus avoiding anterior decompression, if unnecessary. On the other hand, subjects with symptomatic basilar invagination associated with atlanto-axial instability require treatment by reduction and posterior fixation, accepting that, in some instances, an anterior decompression may be indicated for persisting the compression with symptoms. The same option applies to children with CM1 associated with CVJ instability where, in addition, the posterior fossa decompression can be added. It was widely agreed that there is no indication for CVJ fixation in CM1 children if CVJ instability or hypermobility is not documented. Similarly, there was complete agreement to select the treatment (e.g., C0–C3 fixation versus C1–C2 fixation) based on the anatomical condition and the instability and not on the personal belief of the surgeon.

On these grounds, some questions were devoted to the definition (question 4) and the diagnostic work-up of CVJ instability (questions 5, 8, 9). The proposed definition includes also the clinical findings because instability is not ever easy to demonstrate radiologically. A good agreement was achieved about the need to perfect the diagnosis of CVJ instability with dynamic 3D CT scan (thus, not only by dynamic X-rays) and to carefully investigate the course of the vertebral artery and the volume and morphology of the occipital squama and cervical vertebrae prior to surgery. This appears particularly pertinent in children, where the anatomical conditions may vary significantly according to age. For the “simple” diagnosis, on the other hand, dynamic X-rays and MRI are enough (so that the load of CT-related X-rays is avoided).

#### Section 3—Isolated/non-CM1 pediatric syringomyelia

##### Part A: Differential diagnosis (Table 7)

As shown, syringomyelia is frequently associated with CM1 and it is a discriminating factor influencing the indication for surgery and the assessment of outcome [75–77]. Its management is strictly related to the management of CM1. On the other hand, non-CM1 syringomyelia, which is likewise frequent, deserves a separate dissertation because of its varied etiology and management. In this context, the first crucial step to be addressed is the differential diagnosis. Since this type of syringomyelia is often clinically silent, such a differential diagnosis is obtained radiologically. A general agreement was expressed on the need for a whole spinal cord MRI to rule out associated anomalies (namely, but not only, spinal dysraphisms). A high agreement was also reached on the need for contrast medium administration to rule out associated tumors (or vascular malformations). Similarly, MRI with CISS-sequences or, in very selected cases, myelo-CT scan can be considered useful to detect small arachnoid cysts or the signs of arachnoiditis, although the latter diagnosis may be hard to obtain and the sensitivity of these techniques is not absolute. Finally, the need for dynamic studies in case of suspected CVJ or spine instability was reaffirmed.

**Table 7:** Isolated/non-CM1 syringomyelia in children: differential diagnosis, surgical indications, and techniques

**Differential diagnosis:** In children with non-Chiari syringomyelia, a painstaking diagnostic assessment should include:		
1	Whole neuraxis MRI to diagnose an associated dysraphism (tethering of the medulla at any level due to split cord malformation, limited dorsal myeloschisis, retained medullary cord, terminal myelocystocele, conus lipomas, thickened, and fatty filum with a low-lying conus (below L3))	Agreement: 100%
2	Contrast-enhanced MRI to diagnose spinal cord tumor	Agreement: 94.1%
3	Dynamic studies to diagnose spinal cord (congenital or acquired) instability	Agreement: 82.4%
4	CISS-sequence MRI and/or myelo-CT to diagnose associated arachnoidal cysts or arachnoiditis.	Agreement: 81.8%
**Surgical indications—results**		
5	Asymptomatic isolated syringomyelia should be followed up, clinically and radiologically	Agreement: 100%
6	In case of increasing or symptomatic syringomyelia (neurological/neurophysiological deterioration) surgery is indicated	Agreement: 97.1%
7	In case of isolated syringomyelia with progressive scoliosis, surgery for syringomyelia is indicated.	Agreement: 88.2%
**Surgical options—results**		
8	In case of syringomyelia-associated dysraphism, a de-tethering procedure is indicated	Agreement: 81.8%
9	In case of associated spinal cord tumor, its removal is indicated to cure the syringomyelia	Agreement: 100%
10	In case of symptomatic/evolutive syringomyelia secondary to arachnoiditis due to previous trauma and/or surgery, adhesiolysis is indicated	Agreement: 100%
11	In case of symptomatic/evolutive syringomyelia after failure of all the previous treatments (de-tethering, tumor removal, or adhesiolysis) a spino-peritoneal, spino-subarachnoidal, or spino-pleural shunt may be performed	Agreement: 90.9%

##### Part B: Surgical indications (Table 7)

As for CM1, there is no reason to operate on children with asymptomatic, isolated, and stable syringomyelia (universal agreement), based on the low reported risk of evolution [35, 78–80]. In this instance, it is advised to perform a clinical and radiological follow-up, according to the characteristics of each patient. On the other hand, in case of appearance of symptoms and/or clear radiological progression and/or neurophysiological deterioration, surgery is indicated. It is worth noting that, in pediatric cases, scoliosis is considered a possible marker of syringomyelia progression [13]. Of course, to be considered an indication for surgery, the scoliosis progression should be associated with a large syrinx (not with idiopathic hydromyelia, which is commonly coupled with scoliosis).

##### Part C: Surgical technique options (Table 7)

This conclusive part addresses the surgical options according to the different settings possibly associated with non-CM1 syringomyelia. The need to perform treatment directed according to etiology is strongly confirmed. As a consequence, syringomyelia associated with spinal dysraphism should be treated by de-tethering of the spinal cord, while that related to spinal cord tumors by excision of the tumor. Similarly, the challenging syringomyelia resulting from post-traumatic or post-surgical arachnoiditis should be treated by lysis of the adherences when symptomatic or progressive. In the case of post-traumatic/post-surgical arachnoiditis, the surgical results may be disappointing; therefore, lysis should be planned only in case of clearly symptomatic patients. These results sound quite obvious; however, they definitively affirm the important concept that a direct treatment of syringomyelia is not routinely necessary nor advised. Indeed, a direct approach (e.g., shunting) is required only in case of failure of the etiologic treatment [81].

## Conclusions

The consensus process on the controversial topics in children was fruitful and allowed the authors to get some results that can be used as a base for future studies looking for recommendations or guidelines. They can be summarized as follows:

1. The old definition of CM1 is still accepted, although the definition based on level of descent in millimeters alone has little clinical correlation. Attention must be paid not only on the degree of tonsillar descent but also to the morphology and volume of the posterior fossa. Transient tonsillar descent resulting from remitting primary diseases is not a CM1. Syringomyelia is considered if large and/or progressing and/or symptomatic (thin hydromyelia is a para-physiological finding);
2. No surgical indications are recognized in asymptomatic children with CM1 but without syringomyelia;
3. To date, no etiological relationship has been found between CM1 and epilepsy or autism;
4. Asymptomatic children should remain in contact with their physicians until the end of their growth. If needed, clinical and radiological controls are scheduled according to the characteristics of each patient;
5. Symptomatic subjects benefit from a multidisciplinary team to rule out symptoms/signs unrelated to CM1 (e.g., key role of the neurologist in case of migraine-like headache). Some tools (polysomnography, SSEPs, MEPs) are useful in selected, doubtful cases;
6. In CM1 with associated hydrocephalus, hydrocephalus should be treated first to reduce the intracranial pressure, limiting the posterior fossa decompression to the children with persisting symptoms;
7. CM1 can result from both non-syndromic and syndromic craniosynostoses whose treatment needs to be incorporated into the treatment strategy. Cranial vault surgery may address cerebellar ectopia. The posterior fossa decompression or the posterior distraction should be limited to selected cases;
8. Tethered cord is rarely and sporadically associated with CM1 and its management is independent of CM1. A manifest and symptomatic tethered cord is treated by intradural de-tethering but no effects on CM1 are expected;
9. Bony posterior decompression and duraplasty are both acceptable techniques of foramen magnum decompression. The first offers a very low rate of complications and a short hospital stay but shows a higher risk of persisting symptoms: it can be considered in children without severe symptoms or large syringomyelia. The second one ensures more consistent radiological results but with a higher rate of (CSF-related) complications;
10. The bony decompression has to be focused on a proper opening of the foramen magnum and C1 (no need for large opening of the occipital squama or C2);
11. The key points of duraplasty are to keep unviolated the arachnoid plane and to perform a proper dural closure (no need of tonsils coagulation unless this appears necessary to effect an effective and capacious decompression: e.g., tonsils extending below C2);
12. The surgical failure can be defined by the persistence of symptoms 1 year after surgery. The radiological persistence of CM1/syringomyelia should be interpreted in clinical context before a decision to re-operate;
13. Too small opening and incorrect indication are the main causes of failure of bony decompression alone. Re-operation with a larger decompression and duraplasty are the respective solutions;
14. Arachnoiditis is an important cause of failure of duraplasty. Proper surgical technique, re-operation with arachnoid lysis, and, in very selected cases, IV ventricle stenting are the solutions;
15. Asymptomatic postoperative CSF collection can be managed conservatively, while they should be treated promptly, if symptomatic;
16. CVJ instability or craniovertebral deformity (basilar impression) must be ruled out in any (CM1) patient presenting with atypical clinical and/or radiological findings. Only symptomatic subjects should be treated, using a technique chosen according to the patient’s characteristics. The posterior decompression for CM1 can be added in selected cases;
17. Syringomyelia must be always ruled out in CM1 children by MRI of the whole spinal cord;
18. Asymptomatic, isolated, and stable syringomyelia shows a very low risk of evolution (no operation is indicated in this instance). It may require treatment only if symptomatic and the treatment is etiological. The direct management (shunting) should be considered only after failure of the etiologic one.

## Data Availability

Data is available at the reader’s request.
